# Editorial: Epileptic seizure disorders in animal models: advances in translational approaches

**DOI:** 10.3389/fneur.2024.1414940

**Published:** 2024-04-22

**Authors:** Evgenia Sitnikova, Filiz Onat, Gilles van Luijtelaar

**Affiliations:** ^1^Institute of Higher Nervous Activity and Neurophysiology of Russian Academy of Sciences, Moscow, Russia; ^2^Department of Neuroscience, Institute of Health Sciences, Acıbadem University Istanbul, Istanbul, Türkiye; ^3^Donders Centre for Cognition, Radboud University, Nijmegen, Netherlands

**Keywords:** translational neuroscience, rodent models, genetic models, genetic predisposition, epileptogenesis

Epilepsy is the fourth most common neurological disorder worldwide. Many etiologically relevant animal models of epilepsy have been developed in small rodents (mice and rats) that may help to overcome these challenges. Our Research Topic aims to gain fundamental knowledge from animal models of epilepsy to improve our understanding of the neurobiological etiology of seizures, genetic correlates, and related comorbidities of different types of epilepsy. This is a collection of studies that aim to bridge the translational gap between animal data and clinical trials (Figure 1).

**Figure 1:**
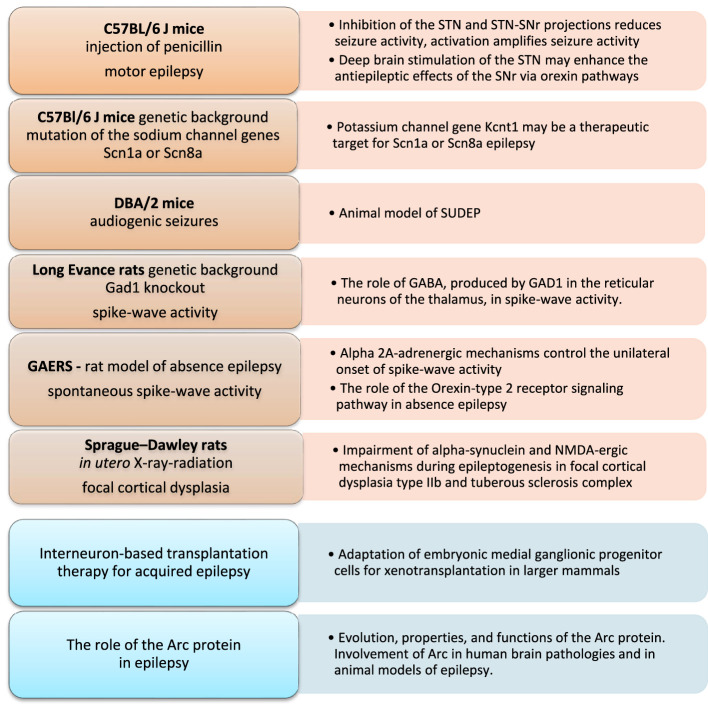
Animal models and approaches to understanding epilepsy as covered in this Research Topic (“*Epileptic seizure disorders in animal models: advances in translational approaches*”). Arc protein, activity-regulated cytoskeleton-associated protein; DBA/2, dilute brown agouti coat color mice; GABA, gamma-aminobutyric acid; GAD1, glutamate decarboxylase 1; GAERS, genetic absence epilepsy rats from Strasbourg; NMDA, N-methyl-D-aspartate; SUDEP, sudden unexpected death in epilepsy.

Topics of interest include the following:

- How can the translational value of animal models be improved?

The review paper by Bosco et al. has focused on sudden unexpected death in epilepsy (SUDEP) as the leading cause of seizure-related premature death, most prevalent in young adults with drug-resistant epilepsy. The authors considered the translational value of animal models and brought together the evidence that dilute brown agouti coat color (DBA/2) mice, which are genetically susceptible to audiogenic generalized seizures, could be regarded as a relevant animal model of SUDEP with a sufficient amount of face validity and perhaps also predictive validity.

Zhang et al. conducted an experimental study in a rat model of focal cortical dysplasia induced by *in utero* X-ray-radiation and compared expression profiles with those obtained in surgical specimens from patients. The study proposes that the epileptogenesis of focal cortical dysplasia type IIb and tuberous sclerosis complex is caused by changes in the expression of α-synuclein, a member of the synuclein family that plays a crucial role in modulating synaptic transmission in the central nervous system, which is associated with an impairment of N-methyl-D-aspartate (NMDA)-ergic mechanisms. Further studies on the modulatory effects of α-synuclein on seizure activity are warranted.

The review paper by Sibarov et al. considered the product of the immediate early gene encoding the activity-regulated cytoskeleton-associated (Arc) protein in the pathogenesis of epilepsy. Arc protein modulates the excitatory-inhibitory balance in mammalian neuronal networks. In active neurons, Arc protein forms retrovirus-like capsids within synapses. After seizures, released Arc particles can accumulate in astrocytes. In their translational approach, the authors focused on rodent models of convulsive and non-convulsive seizures and on genetic models of epilepsy to elucidate the intricate role of Arc in epilepsy. It is proposed that changes in Arc-protein-mediated activity can potentially affect the excitatory-inhibitory balance of the involved neuronal network, either by lowering the seizure threshold and promoting stability or by inducing lability.

- The knowledge gained from rat/mouse strains with a genetic predisposition to epilepsy.

Yavuz et al. conducted an experimental study in the genetic absence epilepsy rat from Strasbourg (GAERS). The authors examined the pro-absence effect of the alpha 2A adrenoreceptor (AR) agonist dexmedetomidine, which was administered systemically in GAERS during the first postnatal month. Their study indicates that the alpha 2A-AR mechanism is involved in the unilateral onset of absence-like seizures in the left cortex in close to 50% of the animals. The unilateral onset does fit with recent theories of the focal onset of an archetypal type of generalized epilepsy.

Toplu et al. used GAERS to investigate the orexin-type 2 receptor (OX2R) signaling pathway in absence epilepsy. The authors discovered that the OX2R agonist had a suppressive effect on spike-wave discharges, as demonstrated by significantly reduced OX2R expression in the cortex and thalamus. This effect may be attributed to the agonist's regulation of the wake/sleep state. The therapeutic potential of targeting OX2R in the treatment of various types of epilepsy, and especially absence epilepsy awaits further research.

- The knowledge gained from knockout and genetically modified rats/mice.

Liu et al. investigated the gamma-aminobutyric acid (GABA)-ergic mechanisms of epileptic activity. They targeted Glutamate decarboxylase 1 (GAD1)- an enzyme involved in the synthesis of GABA and obtained Gad1 knockout rats from the Long-Evans background. The authors propose a functional model in which GABA produced by GAD1 in the reticular thalamic neurons is released outside the synapses and causes inhibition within the reticular thalamic nucleus. Of note, these thalamic neurons are a major source of inhibition of the thalamic-cortical relay cells.

- New genetic technologies to model epilepsy in rats/mice.

The study by Hill et al. was conducted in mice with mutations in the sodium channel genes Scn1a or Scn8a. Mutations in these genes can lead to dysfunction of sodium channels, resulting in increased neuronal excitability and predisposing individuals to seizures, such as in Dravet syndrome and febrile seizures. Both genes are also involved in developmental and epileptic encephalopathies, which represent severe seizure disorders with inadequate treatment options. Their study indicates that a reduction of *Kcnt1* expression is protective in mouse models of *Scn1a* and *Scn8a* epilepsy, suggesting that patients with *SCN1A* and *SCN8A* mutations may benefit from treatment with a *KCNT1* ASO or KCNT1-specific channel blocker.

- Novel treatment options for patients with therapy-resistant epilepsy.

Xue et al. used a penicillin-induced model of motor epilepsy in adult male C57BL/6 J mice and employed optogenetic and chemogenetic approaches to study the involvement of the subthalamic nucleus (STN) and the substantia nigra pars reticulata (SNr) in motor seizures. Inhibition of excitatory neurons in the STN and the projections from the STN to the SNr reduced seizure activity, but activation of these neurons and pathways amplified seizure activity. The authors also concluded that deep brain stimulation of the STN may be a potential treatment for motor epilepsy and may enhance the SNr's antiepileptic effects through orexin pathways.

The review by Righes Marafiga and Baraban discusses interneuron-based transplantation therapy in acquired epilepsy models, such as the well-known pilocarpine model of Temporal Lobe epilepsy. These authors consider a novel strategy that used embryonic medial ganglionic (MGE) progenitor cells to target parvalbumin-expressing and somatostatin-expressing cells in epileptic networks. Most importantly, this represents a true disease-modifying therapy in the form of the selective addition of new inhibitory interneurons to these circuits.

## Author contributions

ES: Writing – review & editing, Writing – original draft, Visualization, Conceptualization. FO: Writing – review & editing. GL: Writing – review & editing.

